# An Approach to Developing Local Climate Change Environmental Public Health Indicators, Vulnerability Assessments, and Projections of Future Impacts

**DOI:** 10.1155/2014/132057

**Published:** 2014-09-30

**Authors:** Adele Houghton, Paul English

**Affiliations:** ^1^Biositu, LLC, 505D W Alabama Street Houston, TX 77006, USA; ^2^California Department of Public Health, Environmental Health Investigations Branch, 850 Marina Bay Parkway, Richmond, CA 94804, USA

## Abstract

Environmental public health indicators (EPHIs) are used by local, state, and federal health agencies to track the status of environmental hazards; exposure to those hazards; health effects of exposure; and public health interventions designed to reduce or prevent the hazard, exposure, or resulting health effect. Climate and health EPHIs have been developed at the state, federal, and international levels. However, they are also needed at the local level to track variations in community vulnerability and to evaluate the effectiveness of interventions designed to enhance community resilience. This review draws on a guidance document developed by the U.S. Council of State and Territorial Epidemiologists' State Environmental Health Indicators Collaborative climate change working group to present a three-tiered approach to develop local climate change EPHIs. Local climate change EPHIs can assist local health departments (LHDs) in implementing key steps of the 10 essential public health services and the U.S. Centers for Disease Control and Prevention's Building Resilience Against Climate Effects framework. They also allow LHDs to incorporate climate-related trends into the larger health department planning process and can be used to perform vulnerability assessments which can be leveraged to ensure that interventions designed to address climate change do not exacerbate existing health disparities.

## 1. Introduction 

In the last two decades, there has been an increasing awareness and interest in the connection between climate change and health, beginning with the publication of the first report of the UN's Intergovernmental Panel on Climate Change in 1991 [1]. Researchers estimate that the global burden of disease attributable to climate change in 2000 was greater than 150,000 deaths (0.3% of global deaths), although this estimate only includes risks from temperature, diarrhea, malnutrition, floods, and malaria [2, 3]. This risk is increasing, and a significant proportion of the health burden due to climate change falls on children [4].

In order to better understand connections between climate change and human health, several groups are developing climate-sensitive indicators of environmental health, including the Council of State and Territorial Epidemiologists (CSTE) [5], the National Environmental Public Health Tracking Program (NEHTP) run by the U.S. Centers for Disease Control and Prevention (CDC) [6], the U.S. Environmental Protection Agency (EPA) [7], the National Research Council [8], the World Health Organization/Europe [9, 10], and the National Climate Assessment and Development Advisory Committee (NCADAC) [11]. Development of these indicators is key for basic surveillance to examine trends and geographic patterns for vulnerability assessments, to help inform public health adaptation strategies, to help project the impacts of climate change on human health, to inform development of dose-response models [12], and to inform proposed public health preventative actions [13, 14].

Although these global and national efforts are important, they focus on regional or county-level indicators that may not be appropriate for the local community level. Many of the datasets used in these efforts aggregate data to a scale that is too large to identify local vulnerabilities or inform local policies. Local health and environment departments take the lead in activities that are directly impacted by the changing climate, such as hazard mitigation planning and response, tracking variations in community vulnerability, and evaluating the effectiveness of interventions designed to enhance community resilience [15]. They also actively participate in collecting data and conducting surveillance on community health concerns that are directly or indirectly affected by climate change [13], such as the possible need for evacuation from the sites of major climate disasters like Hurricanes Katrina, Ike, and Sandy. Local jurisdictions need to develop quantitative measures to track trends in environmental risk, human health outcomes, and population vulnerability to specific climatic events. These measures will also allow local jurisdictions to incorporate climate-related trends into larger department planning processes.

We present an approach designed to help local jurisdictions downscale existing climate and health indicators for use at the local level. Developed by a CSTE workgroup (the State Environmental Health Indicator Collaborative) [16], we present guidelines to establish local climate change environmental health indicators (EPHIs), and, secondarily, a method to incorporate them into vulnerability assessments and health forecasting—placing emphasis on opportunities to partner with external resources at the local, state, and federal levels. We also discuss how developing and using these indicators can support local jurisdiction efforts to provide essential public health services to their communities and help them integrate climate readiness into existing surveillance programs.

## 2. Materials and Methods

### 2.1. Developing Local EPHIs

**Figure 1:**
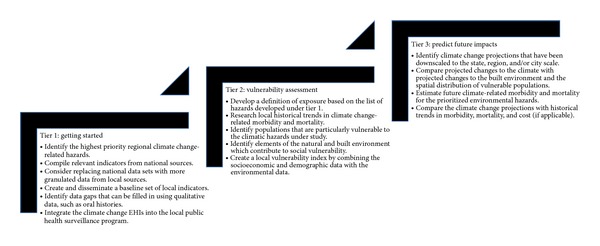
Tiered approach to developing local climate change EPHIs.

Internal capacity, technical expertise, and political mandates vary from one local health department (LHD) to the next. The framework presented in this paper (Figure 1) recognizes the need to tailor engagement around climate and health to the specific needs of individual communities. It, therefore, outlines a tiered approach that meets the needs of both communities that are new to addressing the health effects of climate change, as well as LHDs with sufficient technical capacity and political support to incorporate climate projections into their climate and health planning activities.

### 2.2. Tier 1: Getting Started

The first step in developing local EPHIs for climate change is to identify the highest priority climate change-related hazards in the jurisdiction. Work produced by NCADAC provides an overview of regional climate change-related hazards both according to historical data and future climate projections [17]. Online tools, such as those produced by the Natural Resources Defense Council [18], show historical rates of exposure to climate-related hazards at the state and county levels. Case studies showing examples of the environmental hazards targeted by other US states and cities have been developed by the CDC Climate Ready States and Cities Program [19]. The relevancy of the local set of EPHIs can be maximized by prioritizing the hazards identified in a local climate assessment. For example, LHDs in the Washington, DC, metropolitan area might consider developing EPHIs tracking the health effects of one or more of the following natural hazards, which were identified by the Metropolitan Washington, D.C., Council of Governments as the most significant climate-related threats facing their region: extreme heat, heavy precipitation, severe storms, and sea level rise [20].

In order to become useful decision-support tools, climate change EPHIs at the local level must reflect their political, economic, and social context. It is, therefore, important to balance an initial review of the scientific evidence of local climate-related hazards with existing and planned policies that could influence implementation of interventions tailored to enhance community resilience. For example, a review of the scientific literature and downscaled climate projections might indicate that heat waves and air quality are the most significant environmental hazards in a city. However, if it is located just inland from a highly populated region in a hurricane-prone zone, the political climate might encourage an increased focus on preparing for expected large-scale population displacement in the wake of severe storms.

A local community's hazard mitigation plan may or may not address the health effects of climate change. Many climate change policies in the USA only address mitigation activities (i.e., activities that reduce greenhouse gas emissions [21], such as increasing building energy efficiency or transitioning the city's fleet of vehicles to hybrids). However, even if they do not directly address public health programs, they still indicate policymakers' priorities and objectives. It is, therefore, important to prioritize policy and intervention indicators in the climate change EPHI system with cobenefits for both climate change mitigation and community health.

Initial EHI selection should first involve examination of the state and county indicators developed by the NEHTP [6] and CSTE [16], which point to historical weather, health, and policy data related to climate change hazards such as extreme heat events. Data and step-by-step instructions for assembling these indicators can be found at the NEHTP and CSTE websites: http://ephtracking.cdc.gov/ and http://www.cste.org/group/Indicators/. County data has, in many cases, already been compiled nationally and is available at these websites or from individual state climate or tracking programs [22]. The state health department may be able to provide technical assistance or to collaborate with county health departments to compile indicators that are relevant at the local level. While it is preferable to develop local indicators using national, peer-reviewed datasets, it may be necessary to use a local source—such as data collected through a government epidemiology department, a partner agency, or a local university. In some cases, it may even be necessary to collect new datasets; however, this approach can be time consuming and costly. Proxy indicators may be the most time- and cost-effective mechanism for filling data gaps.

Secondly, where possible, these county-level datasets should be replaced withmore granulated data from local sources, because the aggregation level for some datasets may be too broad to inform local policy. For example, the CSTE climate change indicator, positive test results in sentinels and reservoirs [23], is collected at the state level. However, a vector-borne infectious disease like West Nile Virus will likely spread through a state over a period of time rather than exposing all state residents to equal levels of risk from the beginning of the outbreak. It is important for LHDs to know when the first positive test results approach their jurisdiction, so precautionary measures can be initiated [17, 24]. Furthermore, many of the vulnerability and health outcome indicators outlined from NEHTP and CSTE are relevant to other local surveillance activities, such as asthma reduction programs, chronic disease programs, and flood safety programs. If these programs are already tracking an indicator, the national data source can be replaced with the local source in the final set of local EPHIs. This approach will reduce the likelihood of duplication of efforts across agencies and will help integrate climate readiness considerations into core public health services.

Qualitative data, such as oral histories, can help fill data gaps that are still present at this point. Given the challenges associated with data collection at the local level, it is unlikely that quantitative datasets will paint a comprehensive picture either of population vulnerability or of direct links between public health interventions and community resilience. It is, therefore, important to identify opportunities for incorporating qualitative data into the overall tracking program to capture health determinants that might otherwise be overlooked. For example, in 2009, researchers from the National Center for Atmospheric Research conducted semistructured surveys among populations in Phoenix that had been identified using empirical research methods as potentially vulnerable to negative health outcomes during extreme heat events. The survey gathered information about participants' knowledge of attitude toward and experience with heat waves. It also asked where participants received information and where they turned for help during emergencies (both from within their social network and from community resources). The study results showed that several empirical indicators (such as access to air conditioners) that might be assumed to reduce vulnerability did not necessarily align with the lived reality of these populations, because some participants could not afford to run their air conditioners at a level to sufficiently cool their homes. And many of the survey participants were not aware of community resources designed to address that barrier, such as financial assistance with electric bills and air conditioning repairs [25].

The climate change EPHIs can now be integrated into an agency's overall surveillance program. This step will increase the sustainability of the climate change EPHI program by leveraging existing capacity and data sources. It will also raise awareness throughout the LHD and the community about the many ways that climate change impacts population vulnerability and health outcomes.

When completed, this baseline set of local climate change EPHIs can be shared with agency leadership and the general public. Developing accompanying educational material will assist community engagement efforts in reducing vulnerability, particularly among high-risk populations such as the young, the elderly, and populations with low socioeconomic status.

### 2.3. Tier 2: Vulnerability Assessment

Once local EPHIs have been developed and incorporated into the local public health surveillance system, they can be used to locate clusters of vulnerable populations. For example, EPHIs for extreme heat can be mapped to identify hot spots where both the built environment and resident populations demonstrate high levels of vulnerability. Sister departments and external partners, such as research institutions and nonprofit organizations, may already have developed geospatially coded data sets that can be incorporated into the vulnerability assessment. And state health departments may be able to provide technical assistance supporting the geospatial analysis required to develop the vulnerability index. For example, the Florida ESF8 Planning Map viewer (http://gis.doh.state.fl.us/ESF8PlanningMap/) allows users to overlay social vulnerability indices and vulnerable population densities on top of flooding and storm surge models.

In order to develop a vulnerability assessment, a definition of exposure must be set for the climatic hazard based on either national standards or regional best practices (such as a definition of an extreme heat event set by the National Environmental Health Tracking Program [26]). The selected definition should be applied to historical extreme weather events to establish trends in climate change-related morbidity and mortality [27–29].

The next step is to gather evidence from a combination of the public health literature [13, 30] and local research to identify populations most likely to suffer negative health outcomes after exposure to the climatic hazards included in the analysis. The vulnerability assessment will combine this information with elements of the natural and built environment, such as exposure to urban heat islands (for extreme heat events) or exposure to air pollution and deforestation (for air quality indicators). The resulting vulnerability index will combine the socioeconomic and demographic data with built and natural environment data into a cumulative vulnerability index. Examples of methodologies for developing vulnerability indices and methods for sensitivity testing have been developed by English et al. [31], Houghton et al. [32], Jerrett et al. [33], Reid et al. [34], and Tate [35, 36].

### 2.4. Tier 3: Predict Future Impacts

While developing EPHIs and vulnerability indices using historical data provide a necessary baseline for launching the climate and health planning process, historical trends are not sufficient to prepare for the future effects of climate change. Downscaled climate models must be overlaid on the historical data to develop credible scenarios for how risks, exposures, and vulnerabilities are likely to shift spatially and demographically over time.

Regional downscaled climate change projections can be found through the National Climate Assessment [17]. A number of states and regions have also developed their own, more granular, projections [37]. For example, the New York Climate and Health Project combined heat, air quality, and land-use data to predict the health effects of increasing temperatures [38]. This information can be combined with projected changes to the built environment and demographic shifts to update the vulnerability index according to several likely scenarios. Hayhoe et al. [39], Li et al. [40], and Peng et al. [41] present three methodologies for developing heat wave mortality projections, while Deschênes and Greenstone [42] and Nicholls [43] offer two methodologies for predicting excess all-cause mortality attributable to climate change. Comparisons between baseline vulnerability/mortality and future projections should focus on local policy priorities, such as reducing injury and death rates, bolstering economic prosperity, and reducing health disparities.

### 2.5. Climate Change EPHIs in the BRACE Framework and Essential Public Health Services

Local climate change EPHIs, whether based solely on historical data or incorporating climate projections, provide local health departments with an important tool for implementing key steps of the CDC's Building Resilience Against Climate Effects (BRACE) framework (http://www.cdc.gov/climateandhealth/BRACE.htm/). The BRACE framework relies on data inputs such as EPHIs to demonstrate successful completion of each of its five steps. For example, local environmental exposure and population vulnerability indicators are developed during* Step 1, forecasting climate impacts and assessing vulnerabilities*. EPHIs and vulnerability indices developed exclusively using historical data are valuable inputs during this step. Similarly,* Step 2, projecting the disease burden*, mirrors the process of projecting future trends in vulnerability and mortality after overlaying downscaled climate projections on baseline EPHIs and vulnerability indices.* Step 3, assessing public health interventions*, relies on EPHIs to measure the relative success of policies designed to reduce community vulnerability to climate change. EPHIs should be developed to align with the overarching goals developed in* Step 4* of the BRACE framework,* developing and implementing a climate and health adaptation plan*, so that health indicators are tracked alongside other performance measures managed by sister agencies. Finally, by tracking reductions in population vulnerability and mortality to climate change, EPHIs form the quantitative evidence-base for* Step 5, evaluating impact and improving quality of activities*.

In addition to forming the quantitative backbone of a climate and health program, climate change EPHIs can be integrated into a local health department's core organizational framework by supporting delivery of several of the 10 essential public health services (EPHS). For example, climate change EPHIs support* EPHS 1, monitor health status to identify and solve community health problems*, because they are designed to identify trends in exposure of vulnerable populations to climate change-related events. They also support* EPHS 2, diagnose and investigate health problems and health hazards in the community*, by tracking vulnerabilities and outcomes associated with climate change-related hazards. For example, an air quality EPHI will often track asthma rates associated with increases in ozone formation. Community education and outreach programs developed in accordance with* EPHS 3, inform, educate, and empower people about health issues*, may rely on climate change EPHIs to share data about the links between climate and health, the dangers associated with exposure to climatic hazards, and policies that are available to assist neighborhoods in reducing vulnerability to climatic events. EPHI data can be used to develop maps and other infographics aimed at mobilizing community support for climate and health policies and interventions (*EPHS 4, mobilize community partnerships and action to identify and solve health problems*). Visual materials can also be incorporated into interdepartmental and interagency tabletop exercises, such as using a heat vulnerability map and urban forestry/tree canopy map to inform decisions regarding placement of future municipal tree plantings. Climate change EPHIs can also support* EPHS 5, develop policies and plans that support individual and community health efforts*, by identifying areas that have historically sheltered clusters of vulnerable populations. For example, policymakers might prioritize incentives for constructing vegetated roofs in certain areas of town after reviewing vulnerability indices identifying the neighborhoods that are highly vulnerable to both heat and flooding. Similar to* EPHS 5*, EPHIs can support* EPHS 6, enforce laws and regulations that protect health and ensure safety*, by tracking vulnerability and mortality associated with exposure to high risk climatic events. The data generated through the EPHI tracking process can be used to inform assessment of workforce competency, capacity, access to training, and certifications relevant to climate change, supporting delivery of* EPHS 8, assure competent public and personal health care workforce.* Similarly, EPHIs can be used to generate an evidence base for assessments of local climate adaptation policies designed to protect public health, supporting delivery of* EPHS 9, evaluate effectiveness, accessibility, and quality of personal and population-based health services.* Finally, the process of developing local EPHIs will inevitably lead to uncovering data and research gaps, supporting* EPHS 10, research for new insights and innovative solutions to health problems*.

## 3. Results and Discussion 

The role of climate change EPHIs at the local level is to inform and support local political, economic, and social priorities. In this sense, they play a more active role than many indicators developed at the state and federal levels, which are often used to track long-term trends. The framework outlined here offers enough structure to prioritize specific climate change-related hazards within a local EPHI program. But, it also leaves room for local health departments to tailor their climate change EPHIs to reflect the priorities in local, regional, and state climate change policies and to incorporate locally gathered datasets. Many of these policies are compiled on the Center for Climate Strategies (http://www.climatestrategies.us/), the U.S. Conference of Mayors Climate Protection Agreement (http://www.usmayors.org/climateprotection/agreement.htm), and the U.S. EPA State and Local Climate and Energy Program (http://epa.gov/statelocalclimate/) websites.

The local or regional hazard mitigation plan is another important policy document that could be informed by climate change EPHIs. For example, Baltimore, MD, has combined hazard mitigation planning, floodplain mapping, and climate adaptation planning into single disaster preparedness and planning program [44]. In a similar move to integrate climate change into hazard mitigation planning, Santa Cruz, CA, renamed its local hazard mitigation plan the City of Santa Cruz Climate Adaptation Plan [45] and included a chapter outlining the results of a vulnerability assessment that addressed both social equity and health outcomes associated with high risk climate change-related events.

EPHIs have also informed the wider policymaking process in a number of local jurisdictions. For example, the New York City climate change and public health impact assessment report used published information to establish the localized health effects of climate change-related events [46]. Likewise, Multnomah County, OR, has also used the EPHI model to develop a vulnerability assessment of climate change-related hazards [47].

## 4. Conclusions

Health measures are rarely incorporated into local climate change policies. Developing local climate change EPHIs is one way to start tracking population vulnerability and the relative effectiveness of interventions designed to increase resilience. Local climate change EPHIs allow LHDs to incorporate climate-related trends into the larger health department planning process. These metrics can be used to perform vulnerability assessments highlighting health disparities that may be impacted by climate change. When incorporated into the adaptation and hazard mitigation planning process, vulnerability assessments can be leveraged to ensure that interventions designed to address climate change do not further exacerbate existing disparities.

## References

[1] (1992). *Climate Change: The IPCC 1990 and 1992 Assessments*.

[2] McMichael AJ, Butler CD (2004). Climate change, health, and development goals. *The Lancet*.

[4] Sheffield PE, Landrigan PJ (2011). Global climate change and children's health: threats and strategies for prevention. *Environmental Health Perspectives*.

[5] English PB, Sinclair AH, Ross Z (2009). Environmental health indicators of climate change for the United States: findings from the state environmental health indicator collaborative. *Environmental Health Perspectives*.

[6] McGeehin MA, Qualters JR, Sue Niskar A (2004). National environmental public health tracking program: bridging the information gap. *Environmental Health Perspectives*.

[7] U.S. Environmental Protection Agency (2012). *Climate Change Indicators in the United States, 2012*.

[8] National Research Council (2010). *Monitoring Climate Change Impacts: Metrics at the Intersection of the Human and Earth Systems*.

[9] WHO (2010). *Europe Tools for the Monitoring of Parma Conference Commitments: Report of a Meeting*.

[10] World Health Organization Europe (2011). *Monitoring the Implementation of Parma Conference Commitments: Methodological and Organizational Issues*.

[11] U.S. Global Change Research Program *National Climate Assessment: Indicators System*.

[12] Diaz JH (2004). The public health impact of global climate change. *Family and Community Health*.

[13] Frumkin H, Hess J, Luber G, Malilay J, McGeehin M (2008). Climate change: the public health response. *American Journal of Public Health*.

[14] Patz JA, Engelberg D, Last J (2000). The effects of changing weather on public health. *Annual Review of Public Health*.

[15] Maibach EW, Chadwick A, McBride D, Chuk M, Ebi KL, Balbus J (2008). Climate change and local public health in the United States: preparedness, programs and perceptions of local public health department directors. *PLoS ONE*.

[16] Council of State and Territorial Epidemiologists http://www.cste.org/group/Indicators.

[17] National Climate Assessment and Development Advisory Committee (2013). *Third National Climate Assessment Report: Draft for Public Comment*.

[18] Natural Resources Defense Council Climate Change Threatens Health http://www.nrdc.org/health/climate/.

[19] U.S. Centers for Disease Control and Prevention *Climate-Ready States and Cities Initiative*.

[20] Davis M, Campbell A Summary of Potential Climate Change Impacts, Vulnerabilities, and Adaptation Strategies in the Metropolitan Washington Region: a synopsis of lessons learned from the Metropolitan Washington Council of Governments' climate adaptation planning initiatives from 2010–2012.

[21] Bernstein L, Bosch P, Canziani O (2007). *Climate Change 2007: Synthesis Report. An Assessment of the Intergovernmental Panel on Climate Change*.

[22] US Centers for Disease Control and Prevention CDC Tracking Network: State and Local Tracking Portals. http://ephtracking.cdc.gov/showStateTracking.action.

[23] Council of State and Territorial Epidemiologists Positive Test Results in Sentinels and Reservoirs.

[24] Gubler DJ, Reiter P, Ebi KL, Yap W, Nasci R, Patz JA (2001). Climate variability and change in the United States: potential impacts on vector- and rodent-borne diseases. *Environmental Health Perspectives*.

[25] Hayden MH, Brenkert-Smith H, Wilhelmi OV (2011). Differential adaptive capacity to extreme heat: a Phoenix, Arizona, case study. *Weather, Climate, and Society*.

[26] US Centers for Disease Control and Prevention National Environmental Public Health Tracking Network. http://ephtracking.cdc.gov/.

[27] Basu R (2009). High ambient temperature and mortality: a review of epidemiologic studies from 2001 to 2008. *Environmental Health*.

[28] Borden KA, Cutter SL (2008). Spatial patterns of natural hazards mortality in the United States. *International Journal of Health Geographics*.

[29] Knowlton K, Rotkin-Ellman M, Geballe L, Max W, Solomon GM (2011). Six climate change-related events in the United States accounted for about $14 billion in lost lives and health costs. *Health Affairs*.

[30] Balbus JM, Malina C (2009). Identifying vulnerable subpopulations for climate change health effects in the United States. *Journal of Occupational and Environmental Medicine*.

[31] English P, Richardson M, Morello-Frosh R (2013). Racial and income disparities in relation to a proposed climate change vulnerability screening method for California. *International Journal of Climate Change*.

[32] Houghton A, Prudent N, Scott J, Wade R, Luber G (2012). Local public health surveillance of climate change through GEMSS: a web-based visualization tool. *Applied Geography*.

[33] Jerrett M, Su JG, Reid CE (2012). *Mapping Climate Change Exposures, Vulnerabilities, and Adaptation to Public Health Risks in the San Francisco Bay and Fresno Regions*.

[34] Reid CE, O'Neill MS, Gronlund CJ (2009). Mapping community determinants of heat vulnerability. *Environmental Health Perspectives*.

[35] Tate E (2012). Social vulnerability indices: a comparative assessment using uncertainty and sensitivity analysis. *Natural Hazards*.

[36] Tate E (2013). Uncertainty analysis for a social vulnerability index. *Annals of the Association of American Geographers*.

[37] Cooney CM (2012). Downscaling climate models: sharpening the focus on local-level changes. *Environmental Health Perspectives*.

[38] Knowlton K, Lynn B, Goldberg RA (2007). Projecting heat-related mortality impacts under a changing climate in the New York City region. *The American Journal of Public Health*.

[39] Hayhoe K, Sheridan S, Kalkstein L, Greene S (2010). Climate change, heat waves, and mortality projections for Chicago. *Journal of Great Lakes Research*.

[40] Li T, Horton RM, Kinney PL (2013). Projections of seasonal patterns in temperature-related deaths for Manhattan, New York. *Nature Climate Change*.

[41] Peng RD, Bobb JF, Tebaldi C, McDaniel L, Bell ML, Dominici F (2011). Toward a quantitative estimate of future heat wave mortality under global climate change. *Environmental Health Perspectives*.

[42] Deschênes O, Greenstone M (2011). Climate change, mortality, and adaptation: evidence from annual fluctuations in weather in the US. *American Economic Journal: Applied Economics*.

[43] Nicholls N (2009). Estimating changes in mortality due to climate change. *Climatic Change*.

[44] Baltimore Office of Sustainability The Disaster Preparedness and Planning Project. http://www.baltimoresustainability.org/disaster-preparedness-and-planning-project.

[45] Atchison C (2012). *City of Santa Cruz Climate Adaptation Plan, 2012–2017: An Update to the 2007 Local Hazard Mitigation Plan*.

[46] Kinney PL, Shindell D, Chae E, Winston B (2000). Climate change and public health: impact assessment for the NYC metropolitan region. *Climate Change and a Global City: An Assessment of the Metropolitan East Coast Region*.

[47] Lyons-Eubanks K, Davis M, Simon A (2013). *Climate Change and Public Health Preparation Plan: An Assessment of Public Health Impacts of Climate Change and Actions to Protect Our Health*.

